# 36 year old man presenting with pancreatitis and a history of recent commencement of orlistat case report

**DOI:** 10.1186/1475-2891-5-19

**Published:** 2006-08-28

**Authors:** Sarah Napier, Matthew Thomas

**Affiliations:** 1 Bristol Royal Infirmary, Marlborough Street, Bristol, BS2 8HW, UK

## Abstract

**Background:**

Orlistat is an anti-obesity drug licensed in the United Kingdom for 7 years. We present a case of a patient who developed pancreatitis four days after commencing orlistat.

**Case presentation:**

A 36 year old man presented to hospital with acute severe pancreatitis four days after starting a course of Orlistat, a lipase inhibitor used in the treatment of obesity. A diagnosis of drug related pancreatitis was made by exclusion of other causes of pancreatitis; he was a teetotaller, had a normal serum calcium, had no family history of pancreatitis or hyperlipidaemia, no history of trauma and had no evidence of gallstones on Computerised Tomography scan (CT).

**Conclusion:**

Orlistat was the only drug that had been started recently and has been associated with pancreatitis previously. We found no case reports of similar cases, however 99 cases of orlistat related pancreatitis have been reported to the Food and Drug Administration (FDA), but no causative link has been found in clinical trials by the drug company. It is therefore not on the list of possible complications or side effects of the drug.

## Background

Orlistat is an anti-obesity drug licensed in the U.K. for 7 years. We present a case of a patient who developed pancreatitis four days after commencing orlistat.

## Case presentation

A 36 year old man presented to the emergency department with a 24 hour history of central abdominal pain, two episodes of vomiting and loose stool. He had a past history of Type II Diabetes Mellitus, hypertension, asthma and obstructive sleep apnoea. He weighed 130 kg with a Body Mass Index greater than 40.

His current medications were diltiazem, lisinopril, metformin, glicazide and orlistat. The orlistat had been commenced four days previously.

**Figure 1:**
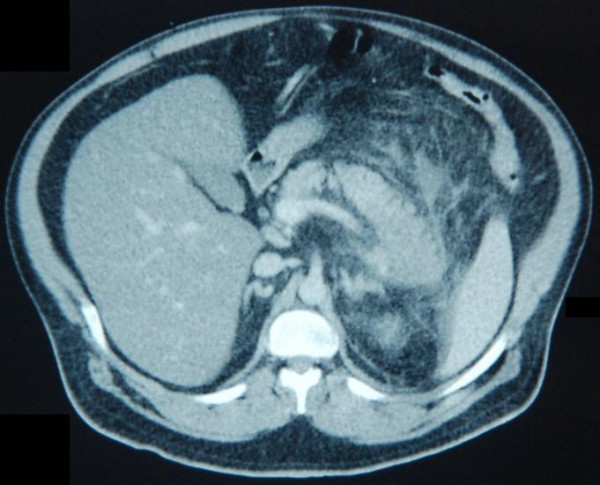
Computerised Tomography of abdomen on admission.

He was pyrexial and on examination was tender in the epigastrium. His initial white cell count was 20 × 10^9^/L and a C reactive protein of more than 300 mg/l, an amylase of 136 iu/l, and a lactate dehydrogenase of 892 iu/l. a recent lipid profile was normal and his corrected calcium was 2.41 iu/l. The initial diagnosis was unclear and a CT scan of his abdomen was organised. This showed appearances of acute pancreatitis affecting the distal body and tail of the pancreas [figure 1]. He was classified as having acute severe pancreatitis using the modified Glasgow Score 1984. Management was the standard of pancreatitis, mainly supportive. Antibiotics were not given. Common causes of pancreatitis were excluded. He was abstinent of alcohol, had a normal serum calcium, had no family history of pancreatitis or hyperlipidaemia, and had no history of trauma. His abdominal CT scan showed no evidence of gallstones. By exclusion the diagnosis of drug induced pancreatitis secondary to orlistat was made.

He was transferred to the intensive care and made good progress. The Medicines Control agency and Committee on Safety of Medicines were informed.

## Discussion

Drug induced pancreatitis accounts for 2% of all cases of pancreatitis [1]. It is a diagnosis of exclusion and should be looked for after ethanol use and cholelithiasis have been excluded. Drug induced pancreatitis usually has a milder clinical course than other causes. Over one hundred drugs have been implicated as causes of pancreatitis, the most common being azathioprine, sulphonamides, sulindac, tetracycline, valproic acid, didanosine, methyldopa, estrogens, furosemide, 6-mercaptopurine, pentamidine, 5-aminosalicylic acid compounds, corticosteroids, and octreotide. Several of these drugs are no longer in common use in the United Kingdom. The mechanisms differ according to different drugs. Some affect the biliary system; others have a direct effect on the pancreas. The diagnosis can be made by a temporal link between a drug and the development of pancreatitis in a patient who does not have other causative factors. In this patient complete abstinence from alcohol and no biliary disease make the diagnosis of drug induced pancreatitis very likely. Orlistat was the only drug that had been started recently and has been associated with pancreatitis previously although no causative link can be shown in clinical trials. The normal amylase was a feature of this case. Amylase is only elevated in 80% of cases of pancreatitis [2]. Orlistat is a pancreatic lipase inhibitor but should have no effect on pancreatic amylase production. CT has long been a well recognised diagnostic tool in pancreatitis [3]

Orlistat is a gastric and pancreatic lipase inhibitor used in obesity management, inhibiting the absorption of fat from the diet and causing a high faecal fat content. It binds to gastric and pancreatic lipase thereby inactivating them; this inhibits the hydrolysis of dietary triglycerides consequently reducing the absorption of monoglycerides and free fatty acids. This causes some unpleasant gastrointestinal side effects including oily discharge, flatulence, faecal urgency, fatty/oily stool, faecal incontinence, abdominal and rectal pain, nausea and vomiting. It is thought that these symptoms cause a behaviour modification in the patient with avoidance of fat-rich foods to avoid the adverse effects. The product information reports abdominal pain as a common and cholelithiasis as a rare complication of orlistat but not pancreatitis. Orlistat has been licensed in the UK since 1998, and gained NICE approval in 2001 [4]. It is licensed for use in the obese population with BMI ≥ 30 kg/m2 or BMI ≥ 27 kg/m2 with other risk factors e.g. hypertension, diabetes or dyslipidaemia. Our patient fitted these characteristics. A literature search found no case reports of associations between orlistat and pancreatitis. We subsequently looked at the licensing of the drug in the U.S, U.K and Canada. The Joint non-prescription drugs advisory committee and endocrinologic and metabolic drugs advisory committee meeting in Maryland in January 23^rd ^2006, discussed Orlistat as a new drug application for Orlistat to become a non prescription drug, had been applied for [5]. They state that there have been 99 raw reports of pancreatitis for orlistat but say that placebo-controlled trials of orlistat in patients treated for 2 years showed no increase in incidence of pancreatitis. The Canadian Adverse Drug Reaction Newsletter reports incidences of pancreatitis secondary to orlistat [6].

Cholelithiasis would seem to be the most obvious link between orlistat and pancreatitis, but this was not present in our case. If orlistat can cause a pancreatitis with normal amylase it is possible that more cases exist but are not fully diagnosed.

## Conclusion

We present a case of acute pancreatitis with normal amylase in a gentleman who had no evidence of biliary disease and who was abstinent of alcohol. The only recent medication change was commencing orlistat four days previously. We suggest that this is a case of orlistat induced pancreatitis. This diagnosis should be considered in patients presenting with abdominal pain, and a normal amylase would not exclude the diagnosis.

## Competing interests

The author(s) declare that they have no competing interests.
